# Identification of mitogen-activated protein kinase docking sites in enzymes that metabolize phosphatidylinositols and inositol phosphates

**DOI:** 10.1186/1478-811X-4-2

**Published:** 2006-01-30

**Authors:** Kevin K Caldwell, Marcos Sosa, Colin T Buckley

**Affiliations:** 1 Department of Neurosciences University of New Mexico Health Sciences Center Albuquerque, NM 87131 USA

## Abstract

**Background:**

Reversible interactions between the components of cellular signaling pathways allow for the formation and dissociation of multimolecular complexes with spatial and temporal resolution and, thus, are an important means of integrating multiple signals into a coordinated cellular response. Several mechanisms that underlie these interactions have been identified, including the recognition of specific docking sites, termed a D-domain and FXFP motif, on proteins that bind mitogen-activated protein kinases (MAPKs). We recently found that phosphatidylinositol-specific phospholipase C-γ1 (PLC-γ1) directly binds to extracellular signal-regulated kinase 2 (ERK2), a MAPK, via a D-domain-dependent mechanism. In addition, we identified D-domain sequences in several other PLC isozymes. In the present studies we sought to determine whether MAPK docking sequences could be recognized in other enzymes that metabolize phosphatidylinositols (PIs), as well as in enzymes that metabolize inositol phosphates (IPs).

**Results:**

We found that several, but not all, of these enzymes contain identifiable D-domain sequences. Further, we found a high degree of conservation of these sequences and their location in human and mouse proteins; notable exceptions were PI 3-kinase C2-γ, PI 4-kinase type IIβ, and inositol polyphosphate 1-phosphatase.

**Conclusion:**

The results indicate that there may be extensive crosstalk between MAPK signaling and signaling pathways that are regulated by cellular levels of PIs or IPs.

## Background

MAPKs catalyze the transfer of the γ-phosphate of adenosine triphosphate (ATP) to serine (S) or threonine (T) residues that precede proline (P) [1,2]; thus, these enzymes are termed proline-directed serine/threonine kinases. Although the sequences ST and TP are sufficient for phosphorylation to occur, the optimal sequence for phosphorylation by a MAPK is PX(S/T)P [1,3]. The majority of cellular proteins contain an SP or a TP sequence, yet, many of these proteins are not MAPK substrates [4], indicating that a mechanism exists for achieving substrate specificity for the MAPKs. This specificity is conferred by the substrate through a docking domain. In addition to underlying specificity, these docking interactions increase the catalytic efficiency of substrate phosphorylation [5-7].

### MAPK docking sites

A MAPK docking site, distinct from the phosphoacceptor site, was first identified in c-Jun [8,9], a c-Jun N-terminal kinase (JNK) substrate; this site was designated the "δ domain". Subsequently, a JNK binding site in the transcription factor ATF-2 [10,11] and a motif termed the "d-box" of Elk-1 that binds ERK2 [4,12] were noted to be similar in sequence to the JNK binding site in c-Jun. Related motifs have been identified in a number of other proteins and have been given various names, including DEJL (docking sites for ERK and JNK, LXL) domain [4], kinase interaction motif (KIM) [13,14], MAPK-docking site [15,16], D box [5,12], D-site [17] and D-domain [6,18-20]. It is important to note that, although these domains were identified based on the ability to bind one or more MAPK, there are differences in the consensus sequences used to identify each of them. For example, MacKenzie et al. [14] proposed a consensus KIM sequence of (V/L)X_2_(R/K)(R/K)X_(3–6)_L, with V, L, R, and K representing the amino acids valine, leucine, arginine and lysine, respectively; Bardwell et al. [16] define a consensus MAPK-binding site sequence of (R/K)_2_X_(2–6)_(L/I)X(L/I), with I representing the amino acid isoleucine; and Kornfeld and colleagues [4] reported two consensus sequences for the DEJL domain: (K/R)X(X/K/R)(K/R)X_(1–4)_(L/I)X(L/I) and (K/R)(K/R)(K/R)X_(1–5)_(L/I)X(L/I). In the present studies we use the term D-domain and the consensus sequences reported by Kornfeld and colleagues [4].

Sharrocks and colleagues [21] report that D-domains are characterized by a cluster of basic residues positioned amino-terminal to an (L/I)X(L/I) motif followed by a triplet of hydrophobic amino acids that precedes a series of proline residues [17,21]. These investigators assessed the role of each of these regions in the binding of ERK2 and p38 to transcription factors, MEF2A, SAP-1, and Elk-1. They determined that mutation of the basic region of the transcription factors reduced their phosphorylation by both phospho-ERK2 and phospho-p38 [21]. This suggests that the basic residues are important for both ERK2 and p38 targeting of MAPK substrates. Mutation of the (L/I)X(L/I) motif (also called the LXL motif) diminished phosphorylation of phospho-ERK substrates, whereas it is not required for phosphorylation of substrates by the MAPK, phospho-p38 [21]. It was also determined that the hydrophobic patch plays an important role in phosphorylation of the substrates by both phospho-ERK and phospho-p38; however, this patch is more important for p38 binding than ERK2 binding. Barsyte-Lovejoy et al. [21] concluded that the proline residues were not important in specificity determination of MAPK substrates. Therefore, the authors hypothesize that the proline residues may play a structural role within the motif.

D-domains can show specificity for families of MAPKs; for example, the Elk-1 D-domain binds JNK and ERK, but not p38 [5,22]; both the SAP-1 and Elk-1 D-domains bind ERK2, whereas the SAP-1, but not Elk-1, D-domain binds p38α [22]. Other D-domains show specificity within a MAPK family; for example, the SAP-1 D-domain binds p38α and p38β, but not p38δ [22]. The D-domain can be positioned either N- or C-terminal to the phosphorylation site [7,12,16,19,23].

A second MAPK docking motif has also been identified: the FXFP motif, or DEF (docking site for ERK, FXFP) motif [4,18,22], where F and P represent the amino acids phenylalanine and proline, respectively. Binding and substrate phosphorylation can occur in the absence of the proline residue [14,18]; however, its presence does increase the effectiveness of the motif [18]. Thus, we chose to include the proline in our searches. The identity of the second (X) residue is highly variable [4,18]. In most, if not all, proteins, the FXFP motif is C-terminal to the phosphorylation site [4,18,19,24]. In general, it appears that the FXFP motif occurs more proximal to a phosphorylation site than is often the case for a D-domain [4,18,20,22,24]. The FXFP motif binds ERK2 and p38α [18,22], but not JNK3 [4] and p38β [4,22]. The FXFP motif and D-domain are each sufficient for MAPK docking; however, when both are present in a protein, they function additively [18,22].

We recently identified a D-domain sequence in PLC-γ1 and provided strong evidence that this sequence mediated an observed interaction between PLC-γ1 and phospho-ERK2 [25]. We have also reported that PLC-γ2, -β1, -β2, and -β4 each have at least one identifiable D-domain, as well co-immunoprecipitate with ERK2 [26]. Based on these observations, we have proposed that MAPK signaling and the metabolism of PIs are integrated. In order to substantiate this hypothesis, we sought to determine whether MAPK docking sites could be recognized in other enzymes that metabolize PIs; additionally, we sought support for extending this hypothesis to include the metabolism of IPs.

### Overview of phosphatidylinositol and inositol phosphate metabolism and signaling

**Figure 1:**
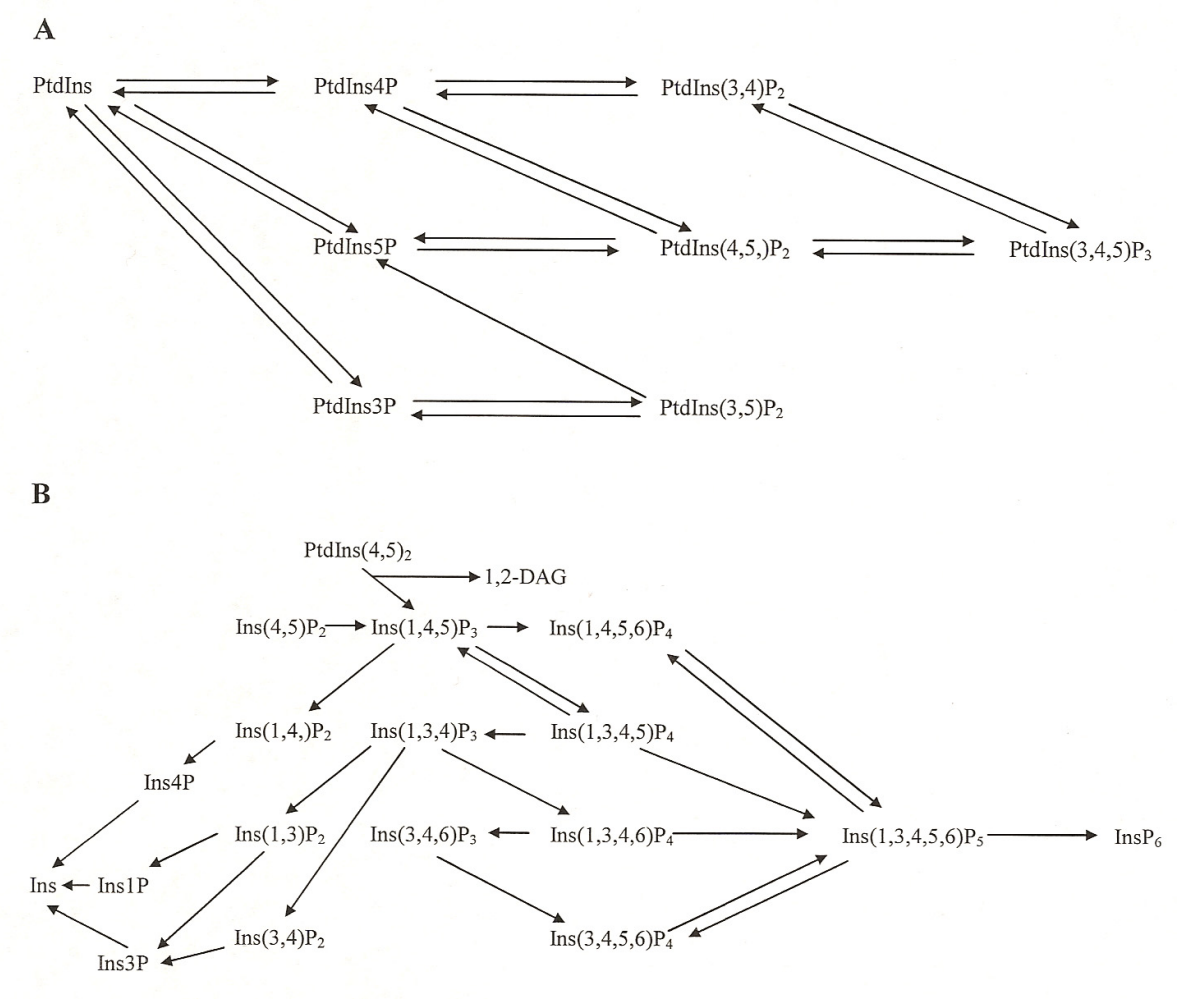
**A: Pathways of metabolism of phosphatidylinositols in animals**. This figure is based on similar figures in Toker [28] and Parker [29] and information provided in the Results and Discussion section. We are not aware of evidence that PtdIns5P is a substrate for a PI 3-kinase, producing PtdIns(3,5)P_2_; therefore, an arrow has not been included for this reaction. **B: Pathways of inositol phosphate metabolism**. The pathways of the metabolism of inositol phosphates in animal cells are shown; additional pathways are present in plants and slime mold [30]. The PLC-catalyzed synthesis of 1,2-diacylglycerol (1,2-DAG) and Ins(1,4,5)P_3 _is also shown. The figure is based on similar figures found in Irvine and Schell [30], Shears [36], and Irvine [37], as well as information provided in the Results and Discussion section.

Eight PIs and more than 20 IPs have been identified [27-30]. Several reviews of the metabolism and cellular roles of these molecules have appeared [27-35]. As the physiologic functions of the PIs and IPs were not a primary focus of the present studies, we will not summarize this information here; instead, the interested reader is directed to the sources cited above; we acknowledge that this is only a partial listing of the reviews that have been written on these subjects. PIs are substrates for a variety of phospholipases, acyl transferases, kinases and phosphatases, while IPs are metabolized by a series of kinases and phosphatases. Of these enzymes, we have limited the scope of the present studies to kinases and phosphatases. In order to assist the reader in understanding the reactions catalyzed by the enzymes which we analyzed, the pathways for the metabolism of PIs and IPs by various kinases and phosphatases are shown in Figures 1A and 1B, respectively. We use the IUPAC-IUB nomenclature for the identification of phosphatidylinositol (PtdIns) and inositol (Ins) phosphates (P): numbers are used to designate the carbon atoms to which phosphate groups are bound and the total number of phosphate groups is designated by a subscript, with the exception that no subscript is employed to designate the presence of a single phosphate group. For example, phosphatidylinositol 4-phosphate is designated PtdIns4P and inositol 1,4,5-trisphosphate is designated Ins(1,4,5)P_3_. For a review of inositol phosphate chemistry the reader is referred to recent articles by Shears [36] and Irvine [37].

## Results and discussion

### Search strategy

We obtained the primary sequences of human and mouse kinases and phosphatases that control the phosphorylation state of the inositol ring in PIs and IPs from GenBank at NCBI and searched these sequences for an FXFP motif and the two consensus sequences for a D-domain reported by Kornfeld and colleagues [4]: (K/R)X(X/K/R)(K/R)X_(1–4)_(L/I)X(L/I) or (K/R)(K/R)(K/R)X_(1–5)_(L/I)X(L/I). We note that this strategy most likely failed to identify all possible sequences that may function as MAPK docking sites. For example, both the human and mouse Class I PI 3-kinase β include the sequence LILRRHGNLFI, which contains a KIM, as defined by MacKenzie et al [16], and a MAPK-docking site, as defined by Bardwell et al. [16], but not a D-domain according to the criteria that we employed. Similarly, the human and mouse Ins(1,3,4)P_3 _5/6-kinase/Ins(3,4,5,6) 1-kinase contain the sequence LCRKRGXEVVQLNL (X is M in human and I in mouse), which fits the consensus sequences for a KIM and a MAPK-binding site, but not a D-domain. We chose to use the criteria of Kornfeld and colleagues based on its successful application in the identification of D-domains in PLC isozymes [25,26]. Although the list that we have compiled is likely to be incomplete, it does serve as a useful first approximation. We also searched each of the enzyme sequences for potential MAPK phosphorylation sites (i.e., (S/T)P sequences) and MAPK optimal phosphorylation sequences, PX(S/T)P. Finally, we note that we did not analyze the sequences of enzymes that control the metabolism of the diphosphorylated IPs.

### Presentation of the data

In order to facilitate the presentation of the data, we separated human from mouse enzymes, kinases from phosphatases, and enzymes in which we identified a D-domain and/or FXFP motif from those in which we did not. Human and mouse kinases having a D-domain and/or FXFP motif are contained in Tables 1 and 3, respectively, whereas human and mouse kinases that do not have either of these sequences are presented in Tables 2 and 4, respectively. Similarly, human and mouse phosphatases containing a D-domain and/or FXFP motif are listed in Tables 6 and 8, respectively, while human and mouse phosphatases that are devoid of these sequences are contained in Tables 7 and 9, respectively.

**Table 1:** Kinases (human) that have an FXFP motif and/or D-domain sequence. The number of potential MAPK phosphorylation sites, sequences fitting the optimal MAPK phosphorylation consensus sequence of PX(S/T)P, and FXFP motifs and D-domain sequences in kinases that use phosphatidylinositols or inositols as substrates are listed in the table; all sequences are for human proteins.

Isozyme	GenBank Accession #	# amino acids	number of (S/T)P sites	Site(s) with the consensus PX(S/T)P sequence	FXFP motif	D-domain motif
*PI 3-kinase*						

*Class I*						
PI 3-kinase α	P42336	1068	6	none	none	^271^KYIRSCIML ^398^RAARLCLSI
PI 3-kinase γ	P48736	1101	13	none	none	^806^KKKPLWL
PI 3-kinase δ	NP_005017	1044	4	none	^585^FSFP	none
*Class II*						
PI 3-kinase C2-α	NP_002636	1686	16	^118^PVTP ^199^PLTP ^202^PATP	none	^238^KNGKARTDLEI
PI 3-kinase, C2-γ	CAA03853	1448	9	none	none	^400^KVSRQCLLTL ^830^KEQKLIKI ^1327^KKPKVQLVI ^1347^KHMKNIHL
*Class III*						
PI 3-kinase Vps34-type	S57219	887	6	none	none	^656^KLLRKENLDL

*PI 4-kinase*						

PI 4-kinase α (PI4K230)	P42356	2044	16	^215^PSSP ^590^PPSP	^1822^FVFP	^15^RRRDAVIAL ^15^RRRDAVIALGI ^1340^KRLREDISI ^1340^KRLREDISIMI ^1429^KRRTLLL ^1429^KRRTLLLSL
PI 4-kinase β (PI4K92)	NP_002642	828	7	none	none	^244^RGTKLRKLIL ^360^KTQRLISELSL ^608^KPYKILVI
PI 4-kinase type II (PI4K55)	NP_060895	479	5	^49^PGSP	none	^435^KDNKSPLHL
PI 4-kinase type II-β (PI4K55)	NP_060793	481	3	none	none	^239^KVGRKFHRIGL

*PIP kinase*						

*Type I PIPK (PI4P 5-kinase)*						
PI4P 5-kinase, type Iα	NO_003548	549	3	^501^PQTP	none	^376^RNSKGERLLL ^379^KGERLLLYI ^379^KGERLLLYIGI
PI4P 5-kinase, type Iβ	NP_003549	540	4	^464^PSTP	none	^335^KSHRGEKLLL
PI4P 5-kinase type Iγ	NP_036530	668	6	^510^PCTP	none	^386^RGERLLLHI ^386^RGERLLLHIGI
*Type III PIPK (PIKfyve; PI3P 5-kinase)*						
PIKfyve	Q9Y2I7	2098	32	^18^PRSP ^21^PTSP ^32^PLTP ^251^PRTP ^581^PFTP ^1524^PPSP	none	^259^KASRNIFL ^700^KNPKILLL ^1981^KMVRDNPLYI

*Ins(1,4,5)P*_3 _*3-kinase*						

Ins(1,4,5)P_3 _3-kinase B	P27987	946	24	^69^PRSP ^164^PRSP ^262^PASP	^56^FLFP	^665^KKKYPWIQL

*Ins(1,3,4,5,6)*_5 _*2-kinase*						

Ins(1,3,4,5,6)_5 _2-kinase	AAM75353	491	5	none	^240^FFFP	none

**Table 2:** Kinases (human) that do not have an FXFP motif and/or D-domain sequence. Kinases that use phosphatidylinositols or inositols as substrates and do not contain an FXFP or D-domain sequence are listed in the table; all sequences are for human proteins. The number of potential MAPK phosphorylation sites and sequences fitting the optimal MAPK phosphorylation in these kinases are also identified.

Isozyme	GenBank Accession #	# amino acids	number of (S/T)P sites	Site(s) with the consensus PX(S/T)P sequence
PI 3-kinase β	NP_006210	1070	3	none
PI 3-kinase, C2-β	O00750	1634	17	none
PIP kinase Type IIα	NP_005019	406	5	^314^PDSP
PIP kinase Type Iiβ	P78356	416	4	^324^PDSP
PIP kinase Type Iiγ	NP_079055	421	1	none
Ins(1,4,5)P_3 _3-kinase A	P23677	461	4	^18^PCSP ^93^PTSP
Ins(1,4,5)P_3 _3-kinase C	NP_079470	683	8	^334^PETP
Ins(1,3,4)P_3 _5/6-kinase/Ins(3,4,5,6)P_4 _1-kinase	NP_055031	414	3	none
Inositol polyphosphate multikinase/Ins(1,3,4,6)P_4 _5-kinase	Q8NFU5	416	5	none

**Table 3:** Kinases (mouse) that have an FXFP motif and/or D-domain sequence. The number of potential MAPK phosphorylation sites, sequences fitting the optimal MAPK phosphorylation consensus sequence of PX(S/T)P, and FXFP motifs and D-domain sequences in kinases that use phosphatidylinositols or inositols as substrates are listed in the table; all sequences are for mouse proteins.

Isozyme	GenBank Accession #	# amino acids	# (S/T)P sites	Site(s) with the consensus PX(S/T)P sequence	FXFP motif	D-domain motif
*PI 3-kinase*						

*Class I*						
PI 3-kinase α	NP_032865	1068	6	none	none	^271^KYIRSCIML ^398^RLARLCLSI
PI 3-kinase γ	NP_064668	1102	12	none	none	^807^KKKPLWL
*Class II*						
PI 3-kinase, C2-α	NP_035213	1509	13	^24^PLTP ^27^PATP ^1374^PFSP	none	^63^KNGKARTDLEI
PI 3-kinase, C2-γ1	NP_997566	1506	12	none	none	^930^KDIKTCHLPL ^1409^KHLKNIHL
PI 3-kinase, C2-γ2	NP_035214	653	3	none	none	^77^KDIKTCHLPL ^556^KHLKNIHL
*Class III*						
PI 3-kinase Vps34-type	NP_852079	887	4	none	none	^656^KLLRKENLDL

*PI 4-kinase*						

PI 4-kinase α (PI4K230)	NP_001001983	2044	16	^215^PSSP ^590^PPSP	^1822^FVFP	^15^RRRDAVIAL ^15^RRRDAVIALGI ^1340^KRLREDISI ^1340^KRLREDISIMI ^1429^KRRTLLL ^1429^KRRTLLLSL
PI 4-kinase β (PI4K92)	NP_780565	801	8	none	none	^232^RGTKLRKLIL ^333^KTQRLISELSL ^581^KPYKILVI
PI 4-kinase type II (PI4K55)	NP_663476	479	5	^49^PCSP	none	^435^KDNKSPLHL

*PIP kinase*						

*Type I PIPK (PI4P 5-kinase)*						
PI4P 5-kinase, type Iα	NP_032872	539	3	^464^PSTP	none	^335^KSHRGEKLLL
PI4P 5-kinase type Iβ	NP_032873	546	5	^498^PQTP	none	^374^RNNKGERLLL ^377^KGERLLLYI ^377^KGERLLLYIGI
PI4P 5-kinase type Iγ	NP_032870	661	5	^509^PCTP	none	^386^RGERLLLHI ^386^RGERLLLHIGI
*Type III PIPK (PIKfyve; PI3P 5-kinase)*						
PIKfyve	NP_035216	2052	31	^18^PRSP ^21^PASP ^32^PLTP ^262^PRTP ^536^PFTP ^1479^PPSP	none	^270^KASRNIFL ^655^KNPKILLL ^1935^KMVRDNPLYI

**Table 4:** Kinases (mouse) that do not have an FXFP motif and/or D-domain sequence. Kinases that use phosphatidylinositols or inositols as substrates and do not contain an FXFP or D-domain sequence are listed in the table; all sequences are for mouse proteins. The number of potential MAPK phosphorylation sites and sequences fitting the optimal MAPK phosphorylation in these kinases are also identified.

Isozyme	GenBank Accession #	# amino acids	# (S/T)P sites	Site(s) with the consensus PX(S/T)P sequence
PI 3-kinase β	NP_083370	1064	4	none
PI 3-kinase δ	NP_032866	1043	3	none
PI 4-kinase type II-β (PI4K55)	NP_083020	445	2	none
PIP kinase Type Iiα	NP_032871	405	4	^314^PDSP
PIP kinase Type Iiβ	Q80XI4	416	3	^324^PDSP
PIP kinase Type Iiγ	NP_473438	421	2	none
Ins(1,4,5)P_3 _3-kinase A	Q8R071	459	5	^18^PCSP ^92^PASP
Ins(1,4,5)P_3 _3-kinase C	NP_853624	678	8	^328^PETP
Ins(1,3,4)P_3 _5/6-kinase/Ins(3,4,5,6)P_4 _1-kinase	NP_766172	419	4	none
Inositol polyphosphate multikinase/Ins(1,3,4,6)P_4 _5-kinase	Q7TT16	396	2	none

**Table 5:** Reactions catalyzed by PIP kinases (based on [50] and [51])

*Type I PIP kinase*
PI → PI5P
PI3P → PI(3,4)P_2_
PI4P → PI(4,5)P_2_
PI3P → → PI(3,4,5)P_3_
PI(3,4)P_2 _→ PI(3,4,5)P_3_

*Type II PIP kinase*

PI3P → PI(3,4,)P_2_
PI5P → PI(4,5)P_2_
PI3P → → PI(3,4,5)P_3_

*Type III*

PI → PI5P
PI3P → PI(3,5)P_2_

**Table 6:** Phosphatases (human) that have an FXFP motif and/or D-domain sequence. The number of potential MAPK phosphorylation sites, sequences fitting the optimal MAPK phosphorylation consensus sequence of PX(S/T)P, and FXFP motifs and D-domain sequences in phosphatases that use phosphatidylinositols or inositols as substrates are listed in the table; all sequences are for human proteins.

Isozyme	GenBank Accession #	# amino acids	# (S/T)P sites	Site(s) with the consensus PX(S/T)P sequence	FXFP motif	D-domain motif
*monophosphatase*						

inositol monophosphatase A1	NP_005527	277	2	none	none	^261^RIAKEIQVIPL

*1-phosphatase*						

Inositol polyphosphate 1-phosphatase	P49441	399	1	none	none	^378^RKRLETFLSL

*3-phosphatase*						

Myotubularin-related protein 1 isoform 1; MTMR1-1	NP_003819	665	3	none	none	^177^KDMRNLRL
Myotubularin-related protein 1 isoform 2; MTMR1-2	NP_789746	568	2	none	none	^177^KDMRNLRL
PI(3,4,5)P_3 _3-phosphatase, PTEN	P60484	403	2	none	^241^FEFP	none

*4-phosphatase*						

Inositol polyphosphate 4-phosphatase, Type Ia	NP_004018	938	7	none	none	^147^RHHRLHLTL
Inositol polyphosphate 4-phosphatase, Type Ib	NP_001557	954	8	none	none	^147^RHHRLHLTL
Inositol polyphosphate 4-phosphatase, Type Iα3	AAK58870	977	7	none	none	^147^RHHRLHLTL
SAC1 poly-phosphatidylinositol phosphatase	AAQH16559	587	3	none	none	^345^KNMRWDRLSI ^348^RWDRLSILL ^461^RTGKRTHLGL ^517^RDWKFLAL ^517^RDWKFLALPI

*5-phosphatase*						

*Group II*						
Inositol polyphosphate 5-phosphatase OCRL-1	Q01968	901	3	none	none	^446^RLLKFDQLNI
Inositol polyphosphate 5-phosphatase OCRL-2	NP_001578	893	3	none	none	^446^RLLKFDQLNI
Synaptojanin 2 (synaptic inositol 1,4,5-trisphosphate 5-phosphatase 2)	O15056	1496	17	^987^PVSP ^1218^PETP ^1249^PLSP	none	^617^RSHRYILL
Sac-domain-containing inositol phosphatase 2 isoform 1; inositol polyphosphate 5-phosphatase F isoform 1	NP_055752	1132	12	^259^PETP	none	^85^KVTKIAVLSL
Sac-domain-containing inositol phosphatase 3	NP_055660	907	5	none	none	^663^KFHKYEEEIDI
Inositol polyphosphate 5-phosphatase, 75 kDa	NP_005531	748	5	none	none	^31^RQSRLLGL ^335^KYAKVKLIRL ^340^KLIRLVGIML
Inositol polyphosphate 5-phosphatase, 75 kDa; Inositol polyphosphate 5-phosphatase B	NP_032411	993	5	none	none	^31^RQSRLLGL ^420^KFVRLVGIML
*Group III*						
Inositol polyphosphate 5-phosphatase, 145 kDa; PI(3,4,5)P_3 _5-phosphatase; SH2-containing inositol 5'-phosphatase 1; SHIP1	NP_005532	1188	18	^286^PESP ^960^PPTP ^968^PISP ^1133^PPTP	none	^348^KSQKFLNKLVI
*Group IV*						
PI polyphosphate 5-phosphatase type IV; Inositol polyphosphate 5-phosphatase E	AAF81404	644	10	^55^PATP ^239^PRSP	none	^79^RLERALSL ^596^RPGRDNIPL ^608^KFDRELYL ^608^KFDRELYLLGI

**Table 7:** Phosphatases (human) that do not have an FXFP motif and/or D-domain sequence. Phosphatases that use phosphatidylinositols or inositols as substrates and do not contain an FXFP or D-domain sequence are listed in the table; all sequences are for human proteins. The number of potential MAPK phosphorylation sites and sequences fitting the optimal MAPK phosphorylation in these kinases are also identified.

Isozyme	GenBank Accession #	# amino acids	# (S/T)P sites	Site(s) with the consensus PX(S/T)P sequence
inositol monophosphatase A2	NP_055029	288	1	none
inositol monophosphatase A3	NP_060283	359	3	none
Myotubularin; MTM1	Q13496	603	3	^586^PTSP ^589^PSSP
Myotubularin-related protein 2; MTMR2	Q13614	643	4	none
Myotubularin-related protein 3; MTMR3	Q13615	1198	9	^582^PTTP
Myotubularin-related protein 6; MTMR6	Q9Y217	621	3	^559^PESP
Inositol polyphosphate 4-phosphatase, Type II	NP_003857	924	7	^485^PPSP
Inositol 1,4,5-trisphosphate 5-phosphatase, Type I	Q14642	412	3	none
Synaptojanin 1 (synaptic inositol 1,4,5-trisphosphate 5-phosphatase 1)	O43426	1575	23	^1090^PATP ^1148^PPSP ^1161^PKSP
Sac-domain-containing inositol phosphatase 2 isoform 2; inositol polyphosphate 5-phosphatase F isoform 2	NP_938144	500	8	none
Sac-domain-containing inositol phosphatase 2 isoform 3; inositol polyphosphate 5-phosphatase F isoform 3	NP_938145	219	1	none
PI(4,5)P_2 _5-phosphatase A; PIB5PA; PIPP	Q15735	1006	26	^148^PRSP ^199^PSTP ^345^PRSP
Skeletal muscle and kidney enriched inositol phosphatase isoform 1; SKIP 1	NP_057616	448	4	^283^PDTP ^356^PSSP
Skeletal muscle and kidney enriched inositol phosphatase isoform 2; SKIP 2	NP_570122	372	4	^207^PDTP ^280^PSSP
Phospholipids-inositol phosphatase; PTEN-like phosphatase; PLIP; PTPM1	AAH20242	201	1	none
Inositol polyphosphate 5-phosphatase 2; SH2-containing inositol 5'-phosphatase 2; SHIP2	JC5765	1258	7	^156^PSSP ^163^PETP ^956^PLTP ^1001^PPSP

**Table 8:** Phosphatases (mouse) that have an FXFP motif and/or D-domain sequence. The number of potential MAPK phosphorylation sites, sequences fitting the optimal MAPK phosphorylation consensus sequence of PX(S/T)P, and FXFP motifs and D-domain sequences in phosphatases that use phosphatidylinositols or inositols as substrates are listed in the table; all sequences are for mouse proteins.

Isozyme	GenBank Accession #	# amino acids	# (S/T)P sites	Site(s) with the consensus PX(S/T)P sequence	FXFP motif	D-domain motif
*monophosphatase*						

inositol monophosphatase A1	O55023	277	2	none	none	^261^RIAKEIEI ^261^RIAKEIEIIPL

*3-phosphatase*						

Myotubularin-related protein 1; MTMR1	NP_058681	669	4	none	none	^181^KDMRNLRL
PI(3,4,5)P_3 _3-phosphatase, PTEN	O08586	403	2	none	^241^FEFP	none

*4-phosphatase*						

Inositol polyphosphate 4-phosphatase, Type Ia	NP_084542	939	7	none	none	^147^RHHRLHLTL
SAC1 poly-phosphatidylinositol phosphatase	CAC20672	587	3	none	none	^345^KNMRWDRLSI ^348^RWDRLSILL ^461^RTGKRTQLGL ^517^RDWKFLAL ^517^RDWKFLALPI

*5-phosphatase*						

*Group II*						
Inositol polyphosphate 5-phosphatase OCRL	NP_796189	900	2	none	none	^445^KLLKFDGLNI
Synaptojanin 2 (synaptic inositol 1,4,5-trisphosphate 5-phosphatase 2)	Q9D2G5	1434	15	^987^PVSP	none	^617^RSHRYILL
Sac-domain-containing inositol phosphatase 3	NP_598760	907	5	none	none	^663^KFHRWEEEIDI
Inositol polyphosphate 5-phosphatase, 75 kDa; Inositol polyphosphate 5-phosphatase B	NP_032411	993	5	none	none	^31^RQSRLLGL ^420^KFVRLVGIML
*Group III*						
Inositol polyphosphate 5-phosphatase, 145 kDa; inositol polyphosphate 5-phosphatase D; SH2-containing inositol 5'-phosphatase 1; SHIP1	NP_034696	1191	12	^962^PPTP ^970^PLSP	none	^352^KSQKFLNKLVI
*Group IV*						
PI polyphosphate 5-phosphatase type IV; Inositol polyphosphate 5-phosphatase E	Q9JII1	647	7	^243^PRSP	none	^83^KLERTLSL ^599^RPGRDNIPL ^611^KFDRELYL ^611^KFDRELYLIGI

**Table 9:** Phosphatases (mouse) that do not have an FXFP motif and/or D-domain sequence. Phosphatases that use phosphatidylinositols or inositols as substrates and do not contain an FXFP or D-domain sequence are listed in the table; all sequences are for mouse proteins. The number of potential MAPK phosphorylation sites and sequences fitting the optimal MAPK phosphorylation in these kinases are also identified.

Isozyme	GenBank Accession #	# amino acids	# (S/T)P sites	Site(s) with the consensus PX(S/T)P sequence
inositol monophosphatase A2	NP_444491	290	1	none
inositol monophosphatase A3	NP_808398	356	2	none
Inositol polyphosphate 1-phosphatase	P49442	396	2	none
Myotubularin; MTM1	Q9Z2C5	603	1	none
Myotubularin-related protein 2; MTMR2;	Q9Z2D1 (AAH63050)	643	4	none
Myotubularin-related protein 3; MTMR3	CAI35186	1159	7	^582^PSTP
Myotubularin-related protein 6; MTMR6	NP_659092	617	3	^555^PETP
Inositol polyphosphate 4-phosphatase, Type Ib (variant)	NP_766559	679	3	none
Inositol 1,4,5-trisphosphate 5-phosphatase, Type I	AAH56341	412	2	none
Synaptojanin 1 (synaptic inositol 1,4,5-trisphosphate 5-phosphatase 1)	Q8CHC4	1574	19	^1145^PPSP ^1158^PKSP ^1443^PNSP
PI(4,5)P_2 _5-phosphatase A; PIB5PA; PIPP	NP_766027	1003	31	^197^PQSP ^200^PSSP ^346^PRSP
Skeletal muscle and kidney enriched inositol phosphatase isoform 1; SKIP 1	Q8C5L6	468	5	none
Phospholipid-inositol phosphatase; PTEN-like phosphatase; PLIP	NP_079852	261	3	none

In the following discussion we use the terms "alternative pair" and "overlapping" in referring to relationships of D-domains. An alternative pair of D-domains has the same amino-terminus and two possible carboxyl-termini: e.g., ^15^RRRDAVIAL and ^15^RRRDAVIALGI in human PI 4-kinase α (Table 1). We use the term overlapping to identify D-domains that have distinct amino-termini and carboxyl-termini, but share a region of sequence: e.g., ^376^RNSKGERLLL and ^379^KGERLLLYI found in human PI4P 5-kinase type Iα (Table 1).

### PI 3-kinase

Phosphatidylinositol 3-kinase (also called phosphoinositide 3-kinase, PtdIns 3-kinase, PI 3-kinase, and PI3K) isozymes catalyze the phosphorylation of the 3-position of the inositol ring of phosphatidylinositols [32]. Three classes (I, II, and III) of PI 3-kinase have been identified [38,39]. These classes are differentiated on the basis of their subunit composition, substrate specificity and mechanisms of regulation. The Class I enzymes are heterodimers of a regulatory subunit, of various sizes, and a catalytic subunit of approximately 110 kDa. Three distinct forms of the catalytic subunit (p110α, p110β, and p110δ) and five forms of the regulatory subunit (p85α, p85β, p55α, p55γ, and p50α) have been identified. The class IB isoform consists of a p101 regulatory subunit coupled to a p100γ catalytic subunit. Class I enzymes catalyze the synthesis of PtdIns3P, PtdIns(3,4)P_2_, and PtdIns(3,4,5)P_3 _[40,41]; PtdIns(4,5)P_2 _may be the preferred substrate *in vivo *[42]. Class II isozymes are monomeric catalytic subunits (α, β, and γ) containing a carboxyl-terminal C2 domain; these enzymes may be referred to as PI3K-C2. PtdIns and PtdIns4P, and under certain conditions PtdIns(4,5)P_2_, are substrates for the Class II PI 3-kinase [39,43]. A single Class III isozyme, which is specific for PtdIns, has been identified [44].

The Class I p110α and p110γ human (Table 1) and mouse (Table 3) proteins contain D-domains, whereas the p110β (Tables 2 and 4) and p110δ (Tables 1 and 4) isozymes do not. Of all the PI 3-kinase sequences that we analyzed, only the human PI 3-kinase δ (Table 1) contains an FXFP motif (^585^FSFP).

The Class II PI3-kinase C2-α contains a D-domain that is conserved in the human (Table 1) and mouse (Table 3) isozymes. PI 3-kinase C2-α was the only PI 3-kinase isozyme in which we found an optimal MAPK phosphorylation sequence (Tables 1, 2, 3, 4). The human and mouse PI 3-kinase C2-α proteins each contain three such sequences; a sequence alignment revealed that two of these (PLTP and PATP) are conserved, while the third, ^118^PVTP and ^1374^PFSP in human and mouse, respectively, is unique. Both the human (Table 1) and mouse (Table 3) PI 3-kinase C2-γ contain D-domains. A sequence alignment of these two proteins revealed that only the last D-domain (^1347^KHMKNIHL in human and ^1409^KHLKNIHL in mouse) aligns; all of the other identified D-domains are unique to one or the other protein. These results indicate the MAPK-dependent regulation of the human and mouse Class II PI 3-kinases may be significantly different and, thus, caution should be exercised when comparing studies on these two proteins.

The human (Table 1) and mouse (Table 3) Class III PI 3-kinase contains a conserved D-domain. Neither of these proteins contains an FXFP motif or an optimal phosphorylation sequence for MAPKs.

### PI 4-kinase

Phosphatidylinositol 4-kinase (PtdIns 4-kinase, PI 4-kinase) catalyzes the phosphorylation of PtdIns to produce PtdIns4P. Subfamilies (Types II and III; or PI4K230, PI4K92 and PI4K55 using the nomenclature of Heilmeyer et al. [45]) of PI 4-kinase have been identified; these posses a conserved C-terminal catalytic domain and diverse N-terminal regulatory domains [45]. Differing physiologic functions have been ascribed to each of the three subfamilies [45-49].

PI4Kα (also called PI 4-kinase 230) contains three alternative pairs of D-domains that are conserved in human (Table 1) and mouse (Table 3) sequences. PI4Kα also contains an FXFP sequence and two optimal MAPK phosphorylation sequences that are conserved in human and mouse proteins. PI 4-kinase β (also called PI 4-kinase 92) contains three D-domains that are conserved in the human (Table 1) and mouse (Table 3) isozymes. The first of these (^232^RGTKLRKLIL) overlaps with an identifiable bipartite nuclear localization sequence motif [45]. The human and mouse PI 4-kinase-β do not contain an FXFP motif or optimal MAPK phosphorylation sequence. The human (Table 1) and mouse (Tabl3 3) PI 4-kinase type II (also called PI 4-kinase 55) contain a conserved D-domain and optimal phosphorylation sequence for a MAPK. Neither protein contains an FXFP motif. The human (Table 1), but not the mouse (Table 4), PI 4-kinase type II-β contains a single D-domain, while neither protein contains a sequence fitting the MAPK optimal phosphorylation sequence or the FXFP motif.

### PIP kinase

Phosphatidylinositol phosphate kinases (PIP kinases, PIPKs) utilize PtdIns3P, PtdIns4P, and PtdIns5P as substrates, catalyzing the synthesis of PtdIns(3,4)P_2_, PtdIns(3,5)P_2_, and PtdIns(4,5)P_2 _(Table 5). These enzymes are also able to catalyze the formation of PtdIns5P and PtdIns(3,4,5)P_3 _(Table 5). Three types (I, II and III) of PIP kinase are defined based on primary sequence, substrate specificity, and subcellular localization [51]. These families are often designated by the reaction that they catalyze most efficiently. Thus, the Type I PIP kinases are also termed PI4P 5-kinase, the Type II PIP kinases are termed PI5P 4-kinases, and the Type III PIP kinases are called PI3P 5-kinases. Three forms (termed α, β, and γ), each with multiple splice variants, of Type I and Type II PIP kinase have been identified [52-55]. It should be noted that the nomenclature for the α- and β-forms of the human and mouse Type I PIP kinase are reversed: that is, the human Type Iα enzyme corresponds to the mouse Type Iβ enzyme, and vice versa.

Each of the Type I isozymes contain at least one identifiable D-domain that was conserved in humans and in mice. In human PI4P 5-kinase Type Iα (Table 1) an alternative pair of D-domains (^379^KGERLLLYI and ^379^KGERLLLYIGI) overlaps with a different D-domain (^376^RNSKGERLLL). This observation raises the intriguing possibility that these sequences are specific for binding of a particular MAPK, or MAPK family, and that there is competition among MAPKs for binding, with the likelihood that a mechanism(s) exists for the regulation of this binding. Further, the MAPK that is bound may affect the kinetics of the signal that is generated and, thus, the downstream response. The identified alternative pair of D-domains in PI4P 5-kinase type Iγ (PIPKIγ) (Tables 1 and 3) may be responsible for binding phospho-ERK1, which has been shown to catalyze the phosphorylation of serine 650 in PIPKIγ [56]. Phosphorylation of serine 650 inhibits the binding of talin to PIPKIγ and may play a role in synaptic neurotransmission and focal adhesion disassembly during mitosis. None of the Type I PIP kinases that we analyzed contains an FXFP motif (Tables 1 and 3). Each of the Type I PIPKs that we analyzed contains an optimal MAPK phosphorylation site that is conserved in human and mouse proteins. In the case of PIPKIγ, this is not serine-650. However, it is possible that other MAPKs are capable of phosphorylating threonine-512 (human)/threonine-511 (mouse).

The Type II isozymes (Tables 2 and 4) were found to be devoid of D-domain sequences and FXFP motifs. However, the Type IIα and Type IIβ isozymes do contain an optimal MAPK phosphorylation sequence that is conserved in human (Table 2) and mouse (Table 4) protein sequences.

The PIKfyve human (Table 1) and mouse (Table 3) sequences each contain six MAPK optimal phosphorylation sequences. This is the most that we found in any of the sequences that we analyzed. These enzymes contain three conserved D-domain sequences, but are devoid of an FXFP motif.

### Ins(1,4,5)P_3 _3-kinase

Ins(1,4,5)P_3 _3-kinase catalyzes the formation of Ins(1,3,4,5)P_4 _from Ins(1,4,5)P_3_. Three forms of Ins(1,4,5)P_3 _3-kinase have been cloned; these forms differ in their molecular mass, regulation by Ca^2^/calmodulin, tissue distribution and intracellular localization [57-64]. All three forms of human (Tables 1 and 2) and both forms of mouse (Table 4) Ins(1,4,5)P_3 _3-kinase contain one or more optimal sequence for MAPK phosphorylation. Human Ins(1,4,5)P_3 _3-kinase B contains both an FXFP motif and a D-domain, whereas the A and C isoforms do not contain either of these two MAPK docking sequences (Tables 1 and 2). Ins(1,4,5)P_3 _3-kinase B, which plays a critical role in T-cell development [65,66], is associated with the endoplasmic reticulum via its N-terminus [57,67]. Thus, it is possible that the MAPK binding to the ^56^FLFP sequence motif and/or phosphorylation of serine-71 within the optimal phosphorylation sequences (^69^PRSP) regulates Ins(1,4,5)P_3 _3-kinase B interaction with the endoplasmic reticulum.

### Ins(1,3,4)P_3 _5/6-kinase/Ins(3,4,5,6) 1-kinase

Wilson and Majerus [68] cloned an Ins(1,3,4)P_3 _5/6-kinase, which was subsequently shown by Yang and Shears [69] to be the same as Ins(3,4,5,6)P_4 _1-kinase. Interestingly, this enzyme also possesses Ins(1,3,4,5,6)P_5 _1-phosphatase activity [70]. Regulation of these latter two reciprocal activities provides a mechanism for tight control of Ins(3,4,5,6)P_4 _levels in cells. The production of Ins(1,3,4,6)P_4 _by Ins(1,3,4)P_3 _5/6-kinase is the rate-limiting step in the synthesis of inositol hexakisphosphate from Ins(1,3,4)P_3 _[71]. We were unable to identify an optimal sequence for MAPK phosphorylation, an FXFP motif or a D-domain in human (Table 2) or mouse (Table 4) Ins(1,3,4)P_3 _5/6-kinase/Ins(3,4,5,6) 1-kinase.

### Inositol polyphosphate multikinase/Ins(1,3,4,6)P_4 _5-kinase

Inositol polyphosphate multikinase catalyzes the formation of Ins(1,3,4,5,6)P_5 _from Ins(1,4,5)P_3 _by phosphorylation of both the 3- and 6-position of the inositol ring [30,36], with phosphorylation of the 3-position possibly preceding that of the 6-position [72]. The enzyme also phosphorylates the 1-position of Ins(4,5)P_2 _to form Ins(1,4,5)P_3 _[72]. Majerus and colleagues [73] reported that *in vitro *the enzyme displays specificity as an Ins(1,3,4,6) 5-kinase. Neither the human (Table 2) nor mouse (Table 4) protein contains an FXFP motif, a D-domain or optimal sequence for MAPK phosphorylation.

### Ins(1,3,4,5,6) 2-kinase

Ins(1,3,4,5,6)P_2 _2-kinase catalyzes the final step in the synthesis of inositol hexakisphosphate from Ins(1,3,4)P_3_, and ultimately from Ins(1,4,5)P_3 _[71]. The human 2-kinase contains an FXFP motif, but does not contain a D-domain (Table 1). We did not identify an optimal sequence for MAPK phosphorylation.

### Inositol monophosphatase

Inositol monophosphatase is a Mg^2+^-dependent enzyme that catalyzes the hydrolysis of Ins1P, Ins3P and Ins4P, as well as several related compounds, but does not hydrolyze Ins2P [74-78]. Inositol monophosphatase has received significant attention as a potential site of action for lithium in the treatment of bipolar disorder. However, recent studies have identified a number of other lithium targets, as well [79].

Three forms of inositol monophosphatase have been identified: A1 (IMPA1), A2 (IMPA2) and A3 (IMPA3). Of these three forms, only inositol monophosphatase A1 contains an identifiable D-domain (Tables 6 and 8). The human and mouse sequences differ by two amino acids in this region resulting in an alternative pair of D-domains in the mouse isoform (and ^261^RIAKEIEI and ^261^RIAKEIEIIPL) and a single D-domain in the human isoform (^261^RIAKEIQVIPL). We did not find an optimal sequence for MAPK-catalyzed phosphorylation or an FXFP motif in any of the human or mouse inositol monophosphatase isozymes.

### 1-phosphatase

Inositol polyphosphate 1-phosphatase is a Mg^2+^-dependent enzyme that hydrolyzes Ins(1,4)P_2 _and Ins(1,3,4)P_3_, and is inhibited by lithium [76,80-82]. Similar to inositol monophosphatase, the 1-phosphatase has received attention as a site of therapeutic action for lithium in the treatment of bipolar disorder [83]. Notably, the human (Table 6), but not the mouse (Table 9), 1-phosphatase contains a D-domain. Neither the human nor mouse enzyme contains an FXFP motif or an optimal sequence for MAPK phosphorylation.

### 3-phosphatase

Myotubularin, which was originally identified as a candidate gene mutated in X-linked myotubular myopathy [84], has been shown to possess PtdIns3P 3-phosphatase activity [85,86]. A number of myotubularin-related (MTMR) proteins have also been identified [87,88]. Myotubularin (also called MTM1), MTMR1, MTMR2, MTMR3, and MTMR6 possess PtdInd(3)P 3-phosphatase activity [89]. In addition to their PtdIns3P 3-phosphatase activity, myotubularin, [90], MTMR2 [91], MTMR3 [92], and MTMR6 [90] also possess PtdIns(3,5)P_2 _3-phosphatase activity; however, it should be noted that Kim et al. [89] reported that myotubularin and MTMR2 do not hydrolyze PtdIns(3,5)_2_. Additionally, myotubularin and MTMR2 have been shown to hydrolyze Ins(1,3)P_2 _[89].

When we analyzed the human (Table 7) and mouse (Table 9) myotubularin protein sequences, we did not find either an FXFP or D-domain sequence. However, the human sequence does contain two optimal MAPK phosphorylation sequences not present in the mouse sequence. We identified a conserved D-domain in the human (Table 6) and mouse (Table 8) MTMR1 protein, but did not find an optimal sequence for MAPK phosphorylation or FXFP motif in either protein. Neither the human (Table 7) nor the mouse (Table 9) MTMR2 has an optimal sequence for MAPK phosphorylation, a D-doman, or an FXFP motif. Although the human (Table 7) and mouse (Table 9) MTMR3 and MTMR6 each contain a sequence fitting the optimal sequence for MAPK phosphorylation, they do not contain an identifiable D-domain or FXFP motif.

PTEN (phosphatase and tensin homologue deleted on chromosome 10) is a PtdIns(3,4,5)P_3 _3-phosphatase [93], as well as Ins(1,3,4,5)P_4 _3-phosphatase [93] and Ins(1,3,4,5,6)P_5 _3-phosphatase [94]. The human (Table 6) and mouse (Table 8) PTEN sequences contain a conserved FXFP sequence, but no identifiable D-domain or optimal MAPK phosphorylation sequence.

### 4-phosphatase

Two forms (Types I and II) of inositol polyphosphate 4-phosphatase have been identified [95-97]. These enzymes cleave the 4-phosphate from Ins(1,3,4)P_2_, Ins (3,4)P_2_, and PtdIns(3,4)P_2_. The Type I 4-phosphatase has been reported to localize to endosomes, where it plays an important role in the generation of PtdIns3P [98]. In growth factor-stimulated cells the Type I 4-phosphatase also localizes to plasma membrane ruffles, where it hydrolyzes PtdIns(3,4,)P_2_, thereby regulating the association of PtdIns(3,4)P_2_-binindg proteins with the plasma membrane [98]. We identified a conserved D-domain in human (Table 6) and mouse (Table 8) Type I 4-phosphatase. However, we did not identify an optimal phosphorylation sequence or an FXFP motif in this isoform. In contrast, we did not identify a D-domain in the human Type II 4-phosphatase (Table 7), although it does contain a consensus MAPK phosphorylation site. Human Type II 4-phosphatase also does not contain an FXFP motif.

The Sac phosphatase domain, a region of sequence homology found in several yeast, plant and animal proteins, can hydrolyze the 3-, 4-, or 5-position phosphate from PIs, although vicinal phosphate groups are resistant to hydrolysis [99,100]; thus, PtdIns3P, PtdIns4P and PtdIns(3,5)P_2 _are substrates, whereas PtdIns(4,5)P_2 _is not. The PtdIns4P phosphatase activity, but not PtdIns3P or PtdIns(3,5)P_2 _phosphatase activities, of mammalian Sac1 complements phenotypic defects observed in yeast having deletions of Sac1p [101], indicating that the 4-phosphatase activity of these proteins is the most important *in vivo*. The human (Table 6) and mouse (Table 8) Sac1 proteins each contain five identifiable D-domains. Two of these D-domains are overlapping sequences (^345^KNMRWDRLSI and ^348^RWDRLSILL) and two are an alternative pair of D-domains (^517^RDWKFLAL and ^517^RDWKFLALPI). Neither the human nor mouse protein contains an optimal phosphorylation site for MAPKs or an FXFP motif.

### 5-phosphatase

The inositol polyphosphate 5-phosphatases are commonly classified on the basis of their substrate specificities [27,50]. In this system of classification, the Group I enzymes hydrolyze the water-soluble compounds Ins(1,4,5)P_3 _and Ins(1,3,4,5)P_4_; the Group II enzymes hydrolyze both water-soluble and lipid substrates (e.g., PtdIns(4,5,)P_2 _and PtdIns(3,4,5)P_3_); the Group III enzymes hydrolyze the 3-phosphate-containing compounds, Ins(1,3,4,5)P_4 _and PtdIns(3,4,5)P_3_; and, the single Group IV enzyme hydrolyzes only the lipid substrates PtdIns(3,4,5)P_3 _and PtdIns(4,5)P_2 _[27,102]. Although we have used this classification system, we note that recent studies have demonstrated that the substrate specificities of several of the identified 5-phosphatases do not fit into this simple system [103,104]. For example, Schmid et al [103] have shown that there are significant differences in the substrate specificities of several "Type II" 5-phosphatases: synaptojannin 1, synaptojanin 2, the gene product responsible for Lowe's oculocerebrorenal syndrome (OCRL), skeletal muscle and kidney enriched phosphatase (SKIP), and INPP5B. Additionally, it should be noted that the *in vitro *and *in vivo *specificities of these enzymes may differ.

We did not identify a D-domain, FXFP motif or optimal MAPK phosphorylation sequence in either the human (Table 7) or mouse (Table 9) Type I 5-phosphatase. Several of the Group II 5-phosphatases (Tables 6 and 8) contain an identifiable D-domain. OCRL is the gene responsible for occulocerebrorenal dystrophy or Lowe's syndrome, when mutated [27]. Both the human (Table 6) and mouse (Table 8) OCRL proteins contain a D-domain, but do not contain an FXFP motif or an optimal sequence for MAPK phosphorylation. Synaptojanin 1 and synaptojanin 2 are neuronal proteins that play a role in synaptic vesicle trafficking. They contain both a 5-phosphatase domain and a Sac phosphatase domain [99]. The 5-phosphatase domain is responsible for the reported PtdIns(4,5)P_2_-hydrolyzing activity of synaptojanins, while the Sac domain of synaptojanins accounts for their ability to also hydrolyze other PIs, such as the PtdIns4P product generated by the action of its 5-phosphatase domain [105]. Both synaptojanin 1 and 2 contain optimal phosphorylation sites for MAPKs. However, only synaptojanin 2 contains a D-domain, which is conserved in the human (Table 6) and mouse (Table 8) proteins. The synaptojanin sequences that we searched do not contain an FXFP motif. The human (Table 6) and mouse (Table 8) 75-kDa inositol polyphosphate 5-phosphatases (inositol polyphosphate 5-phosphatase B) contain two conserved D-domains, but are devoid of an FXFP motif and an optimal sequence for MAPK phosphorylation.

The Group III SH2-containing inositol 5'-phosphatase 1 (SHIP1) is a hematopoietic-specific enzyme that hydrolyzes both PtdIns(3,4,5)_3 _and Ins(1,3,4,5)P_4 _[106]. In addition, SHIP1 is able to hydrolyze the 4-phosphate from PtdIns(4,5) *in vitro*, thereby generating PtdIns5P [107]. The human (Table 6) and mouse (Table 8) SHIP1 contain an identifiable D-domain. The human SHIP1 contains four optimal MAPK phosphorylation sequences; corresponding sequences for two of these are present in the mouse protein, whereas two are unique to the human protein. SHIP1 does not contain a sequence conforming to an FXFP motif. The distribution of SHIP2 is more ubiquitous than is that of SHIP1 [106]. The human SHIP2 is devoid of a D-domain or an FXFP motif (Table 7). It does contain four sequences that fit the optimal phosphorylation sequence for a MAPK. The human (Table 6) and mouse (Table 8) Group IV 5-phosphatase contain a conserved optimal sequence (PRSP) for MAPK phosphorylation; the human protein contains an additional optimal sequence of MAPK phosphorylation (^55^PATP). Both the human and mouse Group IV 5-phosphatase contain an alternative pair of D-domains, as well as two other D-domains; neither contains an FXFP motif.

### Additional sequence analyses

We examined each of the D-domains that we identified to determine if it overlaps with a KIM [14] or fits the MAPK-docking site consensus sequence defined by Bardwell and colleagues [15,16]. We found only one instance of overlap with a KIM: in the human PI 4-kinase α, the sequence ^12^LDERRRDAVIALGI not only contains the alternative pair of D-domains that we identified (Table 1), but also contains a KIM. Examination of the sequences found in Tables 1, 3, 6, and 8 revealed that, in several instances, the D-domain sequence fits the MAPK-docking sequence of Bardwell and colleagues. It should be noted that in several proteins we identified, but did not catalog, one or more sequence that conformed to the MAPK-docking motif of Bardwell and colleagues but did not conform to the more restrictive sequence that we used for a D-domain.

Finally, we also analyzed the sequences of PtdIns synthase (CDP-1,2-diacyl-*sn*-glycerol:*myo*-inositol 3-phosphatidyltransferase) isozymes, which catalyze the production of PtdIns from cytidine diphosphodiacylglycerol and *myo*-inositol. We did not identify either an FXFP or a D-domain sequence in human or mouse PtdIns synthase sequences (GenBank:NP_006310, NP_665695, and NP_620093; data not shown). Further, we identified a MAPK phosphorylation site only in the human isoform 2. These results indicate that the isozymes that catalyze PtdIns synthesis are not likely to be directly regulated by MAPK; however, they do not rule out the possibility that MAPKs may control PtdIns synthesis via an effect on *myo*-inositol (e.g., via an effect on inositol monophosphatase A1) or cytidine diphosphodiacylglycerol levels.

## Conclusion

We found a high degree of conservation of D-domain sequence and location in human and mouse proteins. Notable exceptions were PI 3-kinase C2-γ, PI 4-kinase type IIβ, and inositol polyphosphate 1-phosphatase. For each of these proteins, either the human or mouse protein contained one or more D-domains that were not found in the other, with the differences in PI 3-kinase C2-γ being the most striking.

Other than within subtypes of an isozyme, we found no evidence of sequence conservation of D-domains in enzymes that metabolize PIs and IPs. That is, the primary sequences of the D-domains that we found were quite variable. This indicates that, both within a family and across families of these enzymes, there may be specificity for binding interactions with MAPKs. Detailed studies examining the relative specificities of each of these proteins for individual MAPKs, if they indeed bind MAPKs, and the contribution of individual amino acids within these sequences to MAPK binding, will provide important information for the development of tools aimed at modifying the integration of MAPK and signaling via PIs and IPs.

When D-domains were found in more than one location in a protein, each D-domain had a unique sequence. This is noted because it is not a universal property of protein-protein interaction domains when present in multiples in a protein- e.g., an SH_2_-binding motif is commonly present in multiple copies in docking proteins [108]. Within a protein, it is possible that each D-domain binds a unique MAPK, or set of MAPKs, allowing for interaction with more than one MAPK signaling pathway, and, thus, the regulation of the metabolism of a particular PI/IP by various combinations of stimuli, or the formation of differing combinations of signaling complexes allowing for the generation of various downstream signals. Further, it is also possible that each interaction is independently regulated. Therefore, it is reasonable to think that these D-domains do not simply serve to amplify signaling through a single signaling pathway, but, instead, allow for the integration of multiple MAPK pathways with the metabolism of a specific PI or IP.

The frequency of occurrence of an identifiable FXFP motif in the enzymes that we analyzed was significantly less than that of a D-domain. There were only five enzymes in which we found a motif conforming to the sequence FXFP; four of these were in kinases (PI 3-kinase δ, PI 4-kinase α, Ins(1,4,5)P_3 _3-kinase B, and Ins(1,3,4,5,6) 2-kinase) and only a single one was found in a phosphatase (PTEN). It is noteworthy that of all the enzymes that we analyzed, only PI 4-kinase α and Ins(1,4,5)P_3 _3-kinase B contain both a D-domain and an FXFP sequence. Finally, we found several sequences fitting an FXF motif (data not shown). As noted in the Introduction, the FXF sequence has been reported to be sufficient for MAPK binding, indicating that these sites may also bind MAPKs.

At this time, we can only speculate on the physiologic significance of the presence of MAPK binding domains in enzymes that control the metabolism of PIs and IPs. We have previously shown that phospho-ERK2-dependent phosphorylation of PLC-γ1 opposes tyrosine kinase-dependent activation of PLC-γ1 [25]. Similarly, MAPKs may regulate (either stimulating or inhibiting) the catalytic activity, or specificity, of kinases and phosphatases that are involved in the metabolism of PIs or IPs, and thereby exert regulatory actions on PI- and/or IP-dependent signaling pathways. Intriguing possibilities exist when a kinase and phosphatase are present in the same complex and one or both of them bind a MAPK. For example, the p85 subunit of Class I PI 3-kinase has been reported to form a complex with Type I inositol polyphosphate 4-phosphatase [109], SHIP1 5-phosphatase [110], and Type IV 5-phosphatase [111]. In these complexes, MAPKs may regulate the relative level or turnover of the substrates and products; for example, by enhancing PI3-kinase activity and associated SHIP1 (or Type IV) 5-phosphatase activity, it would be possible to increase PtdIns(3,4)P_2 _levels without increasing PtdIns(3,4,5)P_3 _levels. Other scenarios (e.g., delayed activation kinetics of the associated 5-phosphatase) are also imaginable, resulting in a transient rise in PtdIns(3,4,5)P_3 _with a delayed elevation in PtdIns(3,4,)P_2 _levels. Similarly, complexes of the Type I inositol polyphosphate 4-phosphatase and PI 3-kinase could produce locally elevated levels of PtdIns3P, without elevating PtdIns(3,4)P_2 _levels, or elevate PtdIns(3,4)P_2 _and, with a delay, PtdIns3P. It is also possible that the interaction between a MAPK and a PI/IP kinase or phosphatase may recruit the MAPK to a multimolecular signal transduction complex containing components of pathways that regulate the activity of the MAPK (e.g., binding of a MAPK to PI 3-kinase may act to recruit the MAPK to a growth factor signaling complex containing Ras and a MAPK kinase) or target the MAPK to a particular subcellular localization (e.g., binding to Type I inositol polyphosphate 4-phosphatase may act to target the MAPK to endosomes).

It should be noted that a protein can serve as a MAPK substrate without directly binding the MAPK: c-Jun-bound proteins that lack a JNK binding site can be phosphorylated by JNK [112]. Thus, our inability to identify a MAPK binding site in a protein does not preclude it from being a MAPK substrate. For example, the MAPK optimal phosphorylation site identified in Type IIα PIPK could be phosphorylated by a MAPK bound to Type Iα PIPK, which has been shown to co-immunoprecipitate with Type IIα PIPK [113]. Similarly, although we did not identify a D-domain or FXFP motif in the 3-phosphatase myotubularin, it may be a MAPK substrate when bound to the 3-phosphatase adaptor protein (3-PAP) subunit [114], which has four recognizable D-domains (data not shown). In fact, human myotubularin does contain two optimal MAPK phosphorylation sequences (Table 7), indicating that it may be a MAPK substrate.

PI 3-kinase, Ins(1,4,5)P_3 _3-kinase B, SHIP1 and PTEN have each been proposed to regulate MAPK signaling. In the case of PI 3-kinase, several studies have been published showing that wortmannin and/or LY-294002, which are inhibitors of PI 3-kinase catalytic activity, block the activation of MAPKs by various stimuli. However, to our knowledge, a direct interaction between a PI 3-kinase and a MAPK has not been demonstrated, and the mechanisms underlying the apparent PI 3-kinase-dependent regulation of MAPKs remain speculative. In the case of PI 3-kinase γ, the effect could be mediated by MEK-1, a MAPK kinase which has been shown to be an *in vitro *substrate of PI 3-kinase γ [115]. Wen et al [65] have shown that ERK1/2 activation in response to suboptimal stimulation of thymocytes is dependent on Ins(1,4,5)P_3 _3-kinase B; they propose a model in which Ins(1,4,5)P_3 _3-kinase B-dependent production of Ins(1,3,4,5)P_4 _acts to sequester an Ins(1,3,4,5)P_4_-binding GTPase-activating protein 1 [116], promoting Ras-dependent activation of ERK1/2. SHIP1, which contains an identifiable D-domain, but no FXFP motif, has been shown to be a negative regulator of JNK activation in B cells [117], ERK1/2 activation in the erythropoietin-dependent cell lineAS-E2 [118], and MAPK (ERK1/2, JNK and p38) activation in RAW264.7 macrophages [119]. In the latter case, the action was shown to be independent of the SHIP1 5-phosphatase activity [119].

Interestingly, SHIP2, which does not have an identifiable D-domain or FXFP motif, has been reported to not exert an effect on cellular MAPKs [120,121]. PTEN, which does not contain a D-domain sequence but does contain an FXFP motif, has been reported to inhibit insulin-stimulated ERK1/2 activation in MCF-7 epithelial breast cancer cells [122,123]. Weng et al. [122] concluded that the effect of PTEN is the result of PTEN-dependent dephosphorylation of the insulin receptor substrate 1, and consequent coupling to ERK1/2 activation. In contrast to the studies of Eng and colleagues, Tang et al. [121] reported that short interfering RNA-induced reductions of PTEN expression in 3T3-L1 adipocytes did not affect insulin-dependent signaling to ERK1/2.

In conclusion, there appear to be a plethora of potential sites of crosstalk between MAPK signaling pathways and the enzymes controlling cellular PIs and IPs, and, thus, the signaling pathways that are regulated by the levels of these intracellular signals. We hope that the identification of these sites of signal integration will initiate a series of studies aimed at determining whether these interactions occur *in vivo *and the physiologic relevance of each to cellular responding.

## Competing interests

The author(s) declare that they have no competing interests

## Authors' contributions

Kevin Caldwell was involved in the conception of these studies, collecting and analyzing sequence data, and drafting and revising the manuscript. Marcos Sosa was responsible for collecting and analyzing sequence data, as well as reviewing the manuscript. Colin Buckley was involved in the conception of these studies, collecting and analyzing sequence data, and reviewing the manuscript. All authors have given final approval of the manuscript.
